# Verification of a clinical decision support system for the diagnosis of headache disorders based on patient–computer interactions: a multi-center study

**DOI:** 10.1186/s10194-023-01586-1

**Published:** 2023-05-23

**Authors:** Xun Han, Dongjun Wan, Shuhua Zhang, Ziming Yin, Siyang Huang, Fengbo Xie, Junhong Guo, Hongli Qu, Yuanrong Yao, Huifang Xu, Dongfang Li, Sufen Chen, Faming Wang, Hebo Wang, Chunfu Chen, Qiu He, Ming Dong, Qi Wan, Yanmei Xu, Min Chen, Fanhong Yan, Xiaolin Wang, Rongfei Wang, Mingjie Zhang, Ye Ran, Zhihua Jia, Yinglu Liu, Xiaoyan Chen, Lei Hou, Dengfa Zhao, Zhao Dong, Shengyuan Yu

**Affiliations:** 1 Department of Neurology, The First Medical Center, Chinese PLA General Hospital, Beijing, 100853 China; 2 International Headache Centre, Chinese PLA General Hospital, Beijing, 100853 China; 3 Department of Neurology, The 940Th Hospital of Joint Logistic Support Force of Chinese People’s Liberation Army, Lanzhou, 730050 Gansu China; 4 School of Health Science and Engineering, University of Shanghai for Science and Technology, Shanghai, 200093 China; 5 AffaMed Therapeutics, Suite 4501, Tower A, Guomao, No. 1 Jianguomenwai Avenue, Beijing, 100004 Chaoyang District China; 6 Department of Neurology, First Hospital of Shanxi Medical University, Taiyuan, 030001 Shanxi China; 7 Department of Neurology, The First Affiliated Hospital of Xiamen University, Xiamen, 361003 Fujian China; 8 Department of Neurology, Guizhou Province People’s Hospital, Guiyang, 550002 Guizhou China; 9 Department of Neurology, Wuhan NO.1 Hospital, Wuhan, 430022 Hubei China; 10 Department of Neurology, Second Hospital of Shanxi Medical University, Taiyuan, 030001 Shanxi China; 11 Department of Neurology, Changsha Central Hospital Affiliated to University of South China, Changsha, 410004 Hunan China; 12 Department of Neurology, Tiantai People’s Hospital of Zhejiang Province, Taizhou, 317200 Zhejiang China; 13 Department of Neurology, Hebei General Hospital, Shijiazhuang, 050051 Hebei China; 14 Department of Neurology, Shandong Provincial Hospital Affiliated to Shandong First Medical University, Jinan, 250021 Shandong China; 15 Department of Neurology, The People’s Hospital of Liaoning Province, Shenyang, 110067 Liaoning China; 16 Department of Neurology and Neuroscience Center, The First Hospital of Jilin University, Jilin, 130031 China; 17 Department of Neurology, Jiangsu Province Hospital, Nanjing, 210029 Jiangsu China; 18 Department of Neurology, Dingyuan General Hospital, Chuzhou, 233290 Anhui China; 19 Department of Neurology, The First Affiliated Hospital of Zhengzhou University, Zhengzhou, 450052 Henan China; 20 Department of Neurology, Linyi Jinluo Hospital, Linyi, 276000 Shandong China

**Keywords:** CDSS, Headache, Diagnostic accuracy, Human–computer conversation

## Abstract

**Background:**

Although headache disorders are common, the current diagnostic approach is unsatisfactory. Previously, we designed a guideline-based clinical decision support system (CDSS 1.0) for diagnosing headache disorders. However, the system requires doctors to enter electronic information, which may limit widespread use.

**Methods:**

In this study, we developed the updated CDSS 2.0, which handles clinical information acquisition via human–computer conversations conducted on personal mobile devices in an outpatient setting. We tested CDSS 2.0 at headache clinics in 16 hospitals in 14 provinces of China.

**Results:**

Of the 653 patients recruited, 18.68% (122/652) were suspected by specialists to have secondary headaches. According to “red-flag” responses, all these participants were warned of potential secondary risks by CDSS 2.0. For the remaining 531 patients, we compared the diagnostic accuracy of assessments made using only electronic data firstly. In Comparison A, the system correctly recognized 115/129 (89.15%) cases of migraine without aura (MO), 32/32 (100%) cases of migraine with aura (MA), 10/10 (100%) cases of chronic migraine (CM), 77/95 (81.05%) cases of probable migraine (PM), 11/11 (100%) cases of infrequent episodic tension-type headache (iETTH), 36/45 (80.00%) cases of frequent episodic tension-type headache (fETTH), 23/25 (92.00%) cases of chronic tension-type headache (CTTH), 53/60 (88.33%) cases of probable tension-type headache (PTTH), 8/9 (88.89%) cases of cluster headache (CH), 5/5 (100%) cases of new daily persistent headache (NDPH), and 28/29 (96.55%) cases of medication overuse headache (MOH). In Comparison B, after combining outpatient medical records, the correct recognition rates of MO (76.03%), MA (96.15%), CM (90%), PM (75.29%), iETTH (88.89%), fETTH (72.73%), CTTH (95.65%), PTTH (79.66%), CH (77.78%), NDPH (80%), and MOH (84.85%) were still satisfactory. A patient satisfaction survey indicated that the conversational questionnaire was very well accepted, with high levels of satisfaction reported by 852 patients.

**Conclusions:**

The CDSS 2.0 achieved high diagnostic accuracy for most primary and some secondary headaches. Human–computer conversation data were well integrated into the diagnostic process, and the system was well accepted by patients. The follow-up process and doctor–client interactions will be future areas of research for the development of CDSS for headaches.

**Supplementary Information:**

The online version contains supplementary material available at 10.1186/s10194-023-01586-1.

## Introduction

Headache disorders are amongst the most prevalent medical conditions in neurological clinics. It is estimated that almost three billion people worldwide experience headaches [1]. In China, the estimated 1-year prevalence of primary headache has been reported to be 23.8% [2]. Because of the massive population, headache remains a huge health burden in China, and the substantial increase in headache cases and years lived with disability (YLDs) represent an ongoing challenge [3]. However, the diagnostic accuracy of headache disorders in China is extremely low. A population-based door-to-door survey conducted in China reported correct diagnostic rates of 13.8% for migraines and only 2.6% for tension-type headaches (TTH) [4]. Of individuals with migraines in China, only 13.5% to 18% had previously been diagnosed [5]. There are several possible reasons for this. First, the third edition of the International Classification of Headache Disorders (ICHD-3), which lists more than 200 headache variants, is the current standard for the diagnosis of headache disorders [6]. This classification system is complicated and challenging for neurologists to use, thus contributing to the difficulty of diagnosing and classifying headaches. Second, because of the large number of outpatients in China, consultation time is often very limited. This increases the chance that physicians will miss key clinical information. Third, different types of headache can have similar symptoms, which makes them challenging for physicians to distinguish. Headache diagnoses are strongly influenced by the headache characteristics provided by patients, and this self-reported information is usually inaccurate and incomplete.

Therefore, there is a strong need for increased consultation efficiency and diagnostic accuracy for patients with headaches. To address this, we developed a computerized clinical decision support system (CDSS) to help clinicians diagnose headaches. Our previous research structured a guideline-based CDSS 1.0, and conducted clinical evaluations at a single headache center [7]. The system showed good sensitivity and specificity for diagnosing primary headaches and medication-overuse headaches, and an improved weighted case-based reasoning method was particularly useful for distinguishing between probable migraines and probable TTH [8]. However, only doctors or medical professionals could enter demographic data and headache characteristics into the system, making it relatively time-consuming to use and thus limiting its application.

To address this in the present study, we developed the updated CDSS 2.0, which is unique in that clinical information is acquired through a human–computer conversation conducted on a personal mobile device in an outpatient setting. In this study, we evaluated its diagnostic accuracy for situations in which data were only collected electronically and actual clinical situations in which assessments were conducted at multiple headache centers. In addition, we investigated patient satisfaction regarding the system.

## Methods

### Study design and patients

**Fig. 1:**
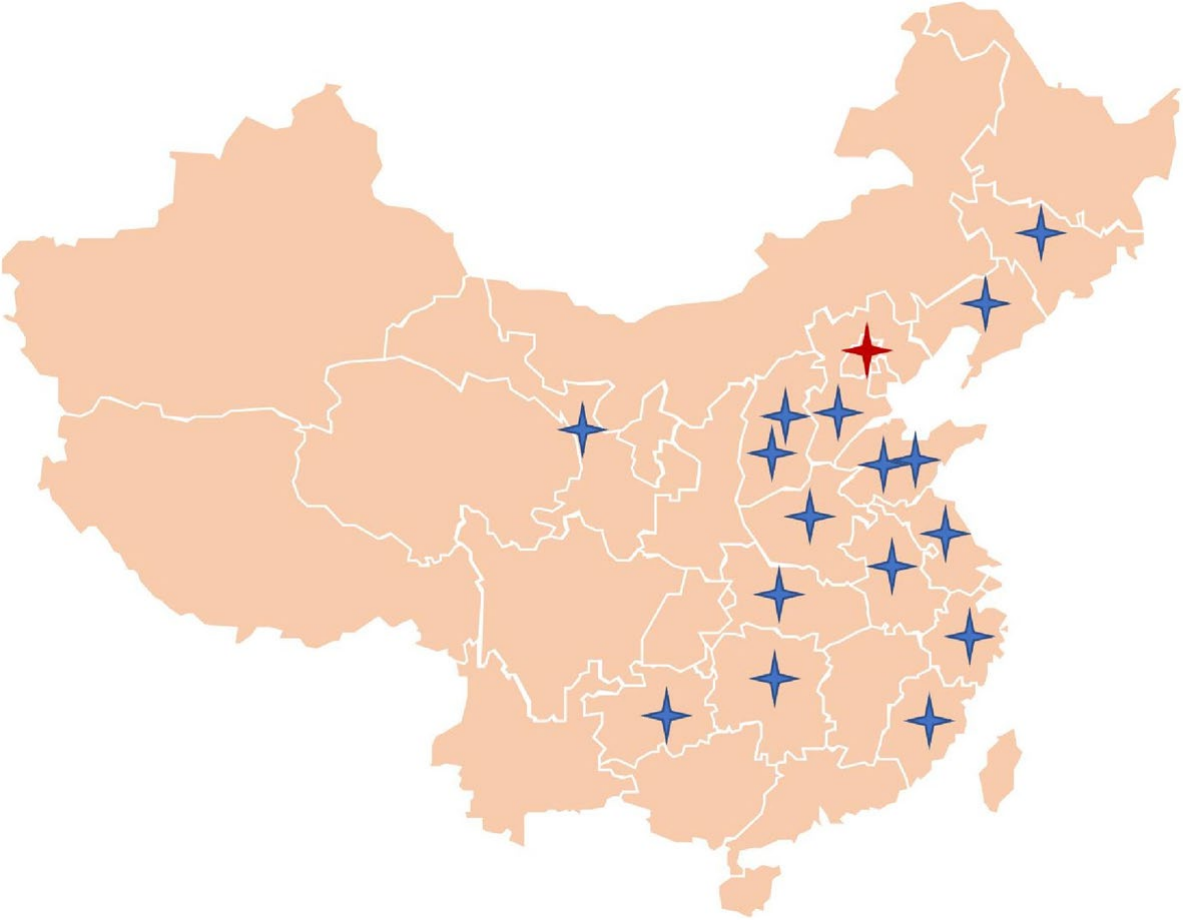
Map of the study centers

This observational study evaluated the diagnostic accuracy of the CDSS 2.0 for headaches as well as patient satisfaction. The system was applied at headache clinics in 16 hospitals in 14 provinces of China (The 940th Hospital of Joint Logistic Support Force of Chinese PLA in Gansu Province, First Hospital of Shanxi Medical University and Second Hospital of Shanxi Medical University in Shanxi Province, The First Affiliated Hospital of Xiamen University in Fujian Province, Guizhou Province People’s Hospital in Guizhou Province, Wuhan NO.1 Hospital in Hubei Province, Changsha Central Hospital Affiliated to University of South China in Hunan Province, Tiantai People’s Hospital in Zhejiang Province, Hebei General Hospital in Hebei Province, Shandong Provincial Hospital Affiliated to Shandong First Medical University and Linyi Jinluo Hospital in Shandong Province, The People’s Hospital of Liaoning Province in Liaoning Province, The First Hospital of Jilin University in Jilin Province, Jiangsu Province Hospital in Jiangsu Province, Dingyuan General Hospital in Anhui Province, The First Affiliated Hospital of Zhengzhou University in Henan Province) were enrolled in the study. The International Headache Center at the Chinese PLA General Hospital served as the main research center (Fig. 1). The participants were voluntarily enrolled at the headache clinics. Patients who experienced headaches were recruited to participate in this study between May 31, 2022, and August 31, 2022, at the selected headache clinics. Patients were asked to read an informed consent form using a mini version of the CDSS 2.0. Anyone who chose not to provide consent had their page withdrawn from the system. Those who consented to participate were asked to complete a patient–computer conversation questionnaire on a personal mobile device. These participants received a normal physician interview and examination in an outpatient setting, and did not receive any incentives for their involvement. Because of the exploratory nature of the study, no formal sample size calculation was performed. The study protocol was approved by the Ethics Committee of the Chinese PLA General Hospital (approval no. S2022–391–01) and met the requirements of the Declaration of Helsinki.

### Design of the patient–computer conversation questionnaire

**Fig. 2:**
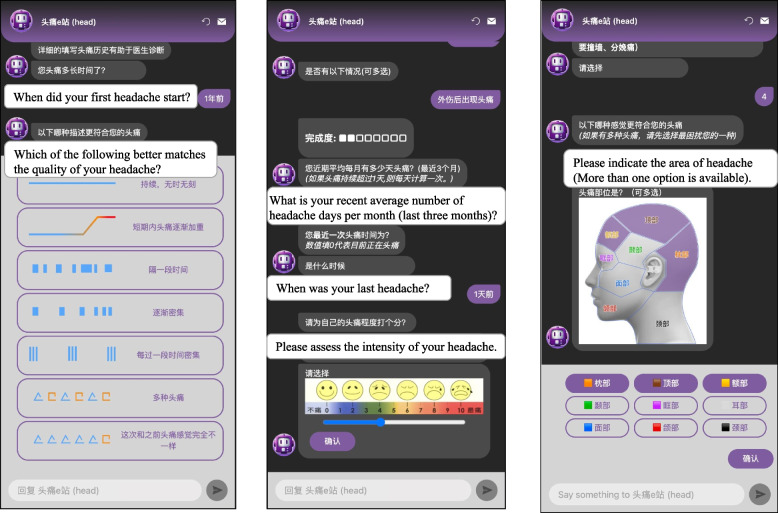
Screenshots of the conversational questionnaire showing examples of information gathered: (**a**) pattern of headache episodes, (**b**) location of headache, (**c**) patient-reported headache severity

First, we designed a web-based software platform to collect clinical information via a conversational questionnaire that was to be completed by outpatients. The patients accessed the questionnaire by scanning a QR code. The questionnaire asked them to describe their headache symptoms via single- or multiple-choice questions. Six experienced neurologists and two headache experts analyzed and modified the questionnaire before deeming it effective for collecting headache-related information (Fig. 2). The questionnaire collected patient demographic data and headache characteristics through a human–computer conversation that took place on a personal mobile device. The questions covered seven predefined themes: demographic information, characteristics and concomitant symptoms of headache, “red-flag” questions, triggers, family history, comorbidities, and previous medical history. The collected demographic data included age, sex, occupation, educational background, height, and weight. Information regarding the characteristics of headache symptoms included the course, disease duration, nature, location, severity, length of each attack, frequency, aura, accompanying symptoms, triggers, alleviative methods, and whether activity aggravated the headache symptoms.

To detect secondary headaches, the questionnaire included “red-flag” questions based on the SNNOOP10 list [9]. These collected information about systemic symptoms including fever, neoplasm history, neurological deficits, sudden or abrupt onset, onset after 50 years, pattern change or recent onset of new headaches, positional headaches, headaches precipitated by sneezing, coughing, or exercise, progressive headaches, eye pain with autonomic features, posttraumatic onset, and so on.

Information about previous treatments, previous examination results (including computed tomography scans of the brain or magnetic resonance imaging), and medical history (including painkiller overuse) was also collected. Measures of anxiety and depression including the Patient Health Questionnaire-9 (PHQ-9) and Generalized Anxiety Disorder Scale (GAD-7) were included in the optional section at the end of the questionnaire.

We anticipated that the questionnaire would be easy for patients to understand and complete, and that it would thus collect more accurate information about headache symptoms. The participants were able to check the degree of completion of the questionnaire at any time during the process. Considering the distinct durations of headache episodes observed in different types of headache condition, we designed the system so that the patients could freely enter numbers and time units. The questionnaire also included multiple choices regarding the nature of headaches and associated symptoms, and each participant was invited to indicate the location of their headaches by touching a picture of a head using their touch screen (Fig. 2). The participants reported headache intensity using a visual analog scale (VAS; 1 representing “no pain” to 10 representing “severe pain”) in which severity was visualized via different crying faces (Fig. 2).

### Verification of the CDSS 2.0

**Fig. 3:**
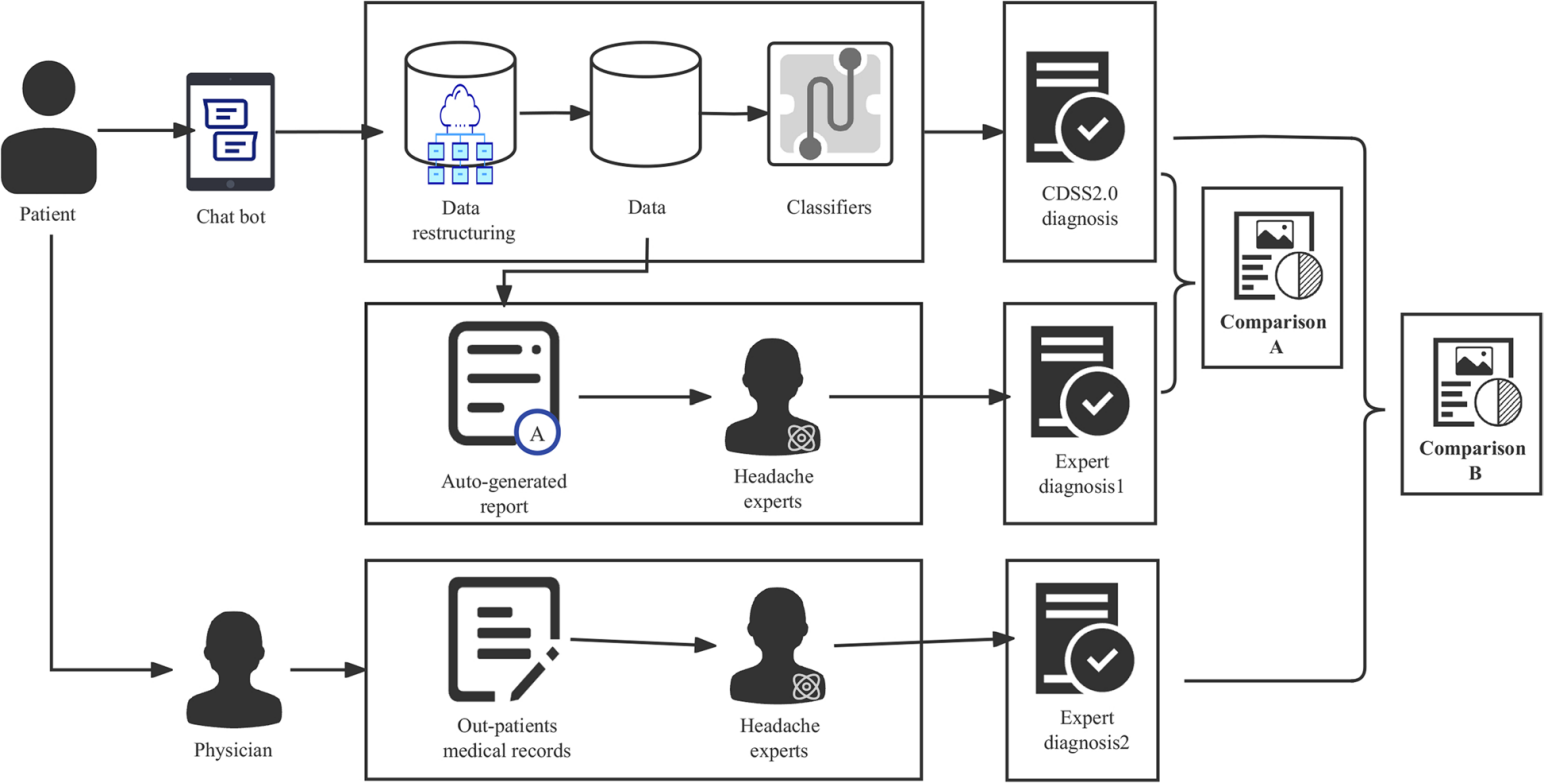
Development process and verification of CDSS 2.0

Researchers at the University of Shanghai for Science and Technology developed the computer-aided diagnosis algorithm, and individuals at AffaMed Therapeutics were responsible for developing the human–computer conversation and the CDSS 2.0 platform (Fig. 3). After information extraction, guideline-based techniques were applied to classify the headache diagnosis based on previously published algorithms developed according to the ICHD-3 [7, 8]. We have designed a diagnostic decision support method suitable for the diagnosis of primary headaches. First, a knowledge representation model for headache diagnosis based on clinical guidelines (ICHD-3) is constructed, and then the model is mapped to a rule set for rule-based reasoning, which is a knowledge base used to diagnose cases with typical symptoms. Moreover, from the simulation of expert's clinical experience, we can build a diagnostic case base based on real clinical dataset for headaches that need differential diagnosis, calculate the weight of each feature in the case base using the method of genetic algorithm, and use case-based reasoning based on weight optimization to find the most similar case in the case base. Case-based reasoning supplements rule-based reasoning. As the patients advanced through the questionnaire, the algorithm presented questions according to their previous answers. This resulted in a different number of respondents per question. If there are any inconsistencies in the patient's answers, the system will automatically identify and repeat the question for confirmation. The categories included migraine, TTH, cluster headache (CH), new daily persistent headache (NDPH), medication-overuse headache (MOH), and neuralgia. Currently, CDSS2.0 could only provide a single diagnosis of headache. For the patient who experienced more than one type of headache, we focused solely on the most troubling one. For patients who meet criteria for both migraine and medication-overuse headache, CDSS2.0 would give the diagnosis of MOH.

The CDSS 2.0 made an automated diagnosis based on the collected information, and simultaneously conveyed a warning to patients with suspicious secondary headache. Two different qualified and experienced headache specialists (XL W and ZD) from the Chinese PLA General Hospital reviewed the clinical information (including auto-generated report, out-patients medical records and preliminary diagnosis from headache centers) of each patient and made two expert diagnoses. Firstly, Expert Diagnosis 1 was made by these two headache specialists based exclusively on the auto-generated report form CDSS 2.0 data. We compared the computerized CDSS diagnosis with Expert Diagnosis 1 to evaluate the diagnostic capability of the CDSS 2.0 system for headache. We also invited the headache specialists to make Expert Diagnosis 2 using a combination of the auto-generated report from CDSS 2.0 and out-patient medical records. The out-patients medical records were obtained from physicians who directly assessed the patients through history-taking and examination, including detailed present history, physical examination, imaging findings, and preliminary diagnosis. To ensure the accuracy of the information, the two headache specialists consulted the physicians for clarification when in doubt. For each patient, we compared the computerized diagnosis with Expert Diagnosis 2 to determine whether the clinical information obtained via the human–computer conversation influenced the diagnostic accuracy.

At the end of the main questionnaire, all patients were asked to complete a perception and satisfaction questionnaire regarding the degree to which they felt the system was easy to understand, useful, and applicable, and to state their general satisfaction with the system. They were also invited to give suggestions on how to improve the system. The questions for this questionnaire were drafted by three headache specialists and discussed with two psychologists until full consensus was reached. The final questionnaire contained eight questions: six questions scored using a 5-point Likert scale, one yes/no question, and one open-ended question. The survey questions are provided in Additional file 1.

### Statistics

SPSS 25.0 software (IBM IncCorp., USA) was used for statistical analysis and Prism 8.0 software was used for mapping. Sensitivity, specificity, and positive and negative predictive values (PPV and NPV, respectively) were calculated for the CDSS diagnoses of headache disorders including migraine with (MO) or without aura (MA), probable migraine (PM), chronic migraine (CM), frequent episodic TTH (fETTH), infrequent episodic TTH (iETTH), probable TTH (PTTH), chronic TTH (CTTH), CH, NDPH, MOH, neuralgia, and unclassified headaches against the two expert diagnoses, respectively. Cohen’s kappa (κ) was calculated to assess agreement between diagnoses. The Kruskal–Wallis test was used to examine the duration of the questionnaire. A 5% level of significance and 95% confidence intervals (CI) were utilized, and data are presented as the mean ± standard error of the mean (SEM).

## Results

### Patient demographic characteristics

In total, 1188 patients with a chief complaint of headache from 16 headache clinics were invited to participate in the study. Of these, 218 patients who declined to participate and 80 patients who did not complete the questionnaire were excluded. Hence, 890 patients agreed to participate in the study and completed the questionnaires.

**Fig. 4:**
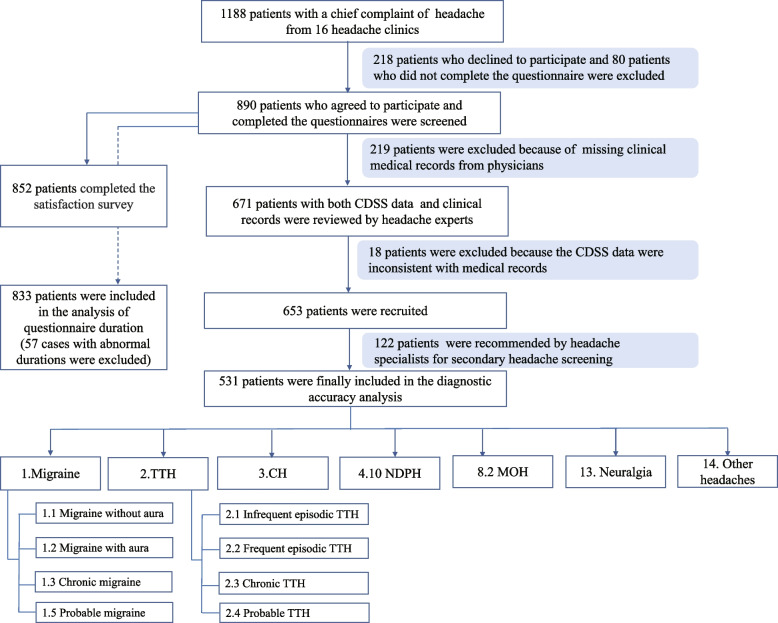
Study flow chart. CDSS: clinical decision support system; TTH: tension-type headache; CH: cluster headache; NDPH: new daily persistent headache; MOH: medication overuse headache

After excluding 219 patients because of incomplete clinical medical records from physicians or missing primary expert diagnosis from 16 headache centers, data from 671 patients were assessed by two headache experts from the Chinese PLA General Hospital. We excluded 18 patients because of contradictory questionnaire data (e.g., the time of onset or frequency of headache were inconsistent between the electronically collected information and clinical records). As a result, 653 patients (247 males and 406 females, age 38.36 ± 14.21 years, range 11–85 years, 92 from The 940th Hospital of Joint Logistic Support Force of Chinese PLA, 63 from First Hospital of Shanxi Medical University, 58 from The First Affiliated Hospital of Xiamen University, 54 from Guizhou Province People’s Hospital, 49 from Wuhan NO.1 Hospital, 45 from Second Hospital of Shanxi Medical University, 44 from Changsha Central Hospital Affiliated to University of South China, 43 from Tiantai People’s Hospital of Zhejiang Province, 42 from Hebei General Hospital, 40 from Shandong Provincial Hospital Affiliated to Shandong First Medical University, 38 from The People’s Hospital of Liaoning Province, 20 from The First Hospital of Jilin University, 19 from Jiangsu Province Hospital, 18 from Dingyuan General Hospital, 15 from The First Affiliated Hospital of Zhengzhou University, 13 from Linyi Jinluo Hospital) were enrolled in the study. Among the patients, 122 were considered highly suspicious of second headache by headache experts, and screening for secondary causes was recommended firstly. Finally, we included 531 patients with primary headaches (including migraine, TTH, CH, and NDPH), MOH, and neuralgia (Fig. 4) in the diagnostic accuracy analysis.

### Questionnaire duration analysis of the CDSS2.0

**Fig. 5:**
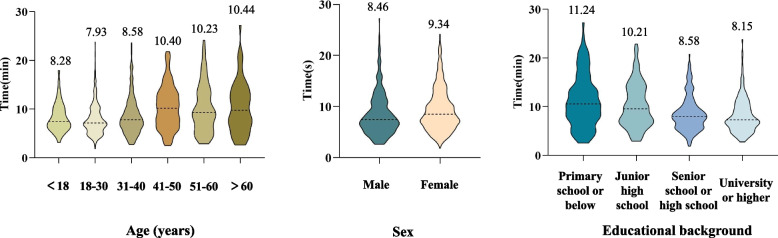
Questionnaire duration for 833 patients according to age, sex, and educational background. The average completion time was shorter in younger (18–30 years) and more highly educated (university degree or above) patients. Males usually took less time to complete the questionnaire than females

The questionnaire included more than 60 questions that collected demographic data regarding the patients and the characteristics of their headaches. Of the 890 patients who provided a complete electronic questionnaire, the average completion time was 10.95 min, and the median was 8.44 min. The questionnaire duration showed a skewed distribution, and these outliers after the 97.5th percentile (29.88 min) in the skewed distribution were excluded. The majority of participants (*n* = 868, 97.5%) completed the questionnaire in less than 30 min. Therefore, we excluded 57 patients with abnormal questionnaire durations, including those whose total duration exceeded 30 min (22 patients) and those who took more than 10 min to complete the basic demographic information Sect. (35 patients). The average completion time of these 22 cases was 65 min, which could not reflect the real time of patients to complete the questionnaire. Ultimately, 833 patients were included in the questionnaire duration analysis. The average completion time was 9.02 min and the median was 8.01 min. The majority of participants (754/833, 90.5%) completed the questionnaire in less than 15 min. When grouped according to age and educational background, the completion time was shorter in younger and more highly educated patients (Fig. 5). Individuals in the 18–30 age group completed the questionnaire relatively quickly (7.93 ± 0.22 min), as did those with a university degree or higher (8.15 ± 0.18 min). Individuals older than 60 years (10.44 ± 0.69 min) and those who had completed primary school or below (11.24 ± 0.52 min) took the longest time to complete the questionnaire. Males (8.46 ± 0.24 min) also took less time than females (9.34 ± 0.18 min).

### Diagnostic accuracy of the CDSS 2.0

Of the 653 recruited patients, 18.68% (122/653) were considered by specialists to require screening for secondary headache. All of these patients were given a warning regarding the risk for a secondary cause according to their answers to the “red-flag” questions in the CDSS 2.0.

Then, for 531 patients, the CDSS 2.0 diagnosis and the two expert diagnoses were compared. The headache specialists made Expert Diagnosis 1 based solely on the auto-generated information. In addition, they verified the diagnoses based on the auto-generated information and the out-patients medical records from physicians, and then made a further diagnosis by combining the two types of information (Expert Diagnosis 2). The distributions of headache type by CDSS 2.0 diagnosis, Expert Diagnosis 1, and Expert Diagnosis 2 are presented in Table 1. Comparison A was performed between the CDSS 2.0 diagnosis and Expert Diagnosis 1, and Comparison B was made between the CDSS 2.0 diagnosis and Expert Diagnosis 2.

**Table 1:** Diagnostic distribution of CDSS 2.0 diagnosis, Expert Diagnosis 1, and Expert Diagnosis 2 for 531 patients

*N* = 531	CDSS2.0 diagnosis	Expert Diagnosis 1	Expert Diagnosis 2
MO	116	129	146
MA	38	32	26
PM	91	95	85
CM	11	10	10
iETTH	11	11	9
fETTH	38	45	44
PTTH	68	60	59
CTTH	24	25	23
CH	10	9	9
NDPH	5	5	5
MOH	28	29	33
Neuralgia	26	45	50
Others	65	36	32

*CDSS* clinical decision support system, *MO* migraine without aura, *MA* migraine with aura, *PM* probable migraine, *CM* chronic migraine, *iETTH* infrequent episodic tension-type headache, *fETTH* frequent episodic tension-type headache, *PTTH* probable tension-type headache, *CTTH* chronic tension-type headache, *CH* cluster headache, *NDPH* new daily persistent headache, *MOH* medication overuse headache

The diagnostic agreement according to Comparison A is presented in Table 2. The CDSS 2.0 correctly recognized 234/266 (87.97%) of patients with migraines. In terms of migraine subtypes, it correctly recognized 115/129 (89.15%) of the patients with MO, 32/32 (100%) of the patients with MA, 10/10 (100%) of the patients with CM, and 77/95 (81.05%) of the patients with PM (Table 2). It also correctly identified 123/141 (87.23%) of the patients with TTH, of which iETTH was diagnosed in 11/11 (100%), fETTH in 36/45 (80.00%), CTTH in 23/25 (92.00%), and PTTH in 53/60 (88.33%). The correct recognition rates for CH and NDPH were 88.89% (8/9) and 100% (5/5), respectively. In addition, the system recognized 28/29 (96.55%) of the patients with MOH. However, it could only correctly recognize 26/45 (57.78%) of the patients with neuralgia.

**Table 2:** Agreement between CDSS 2.0 diagnosis and Expert Diagnosis 1 in Comparison A

CDSS 2.0∖Expert	Total	MO	MA	PM	CM	iETTH	fETTH	PTTH	CTTH	CH	NDPH	MOH	Neuralgia	Others
Total	531	129	32	95	10	11	45	60	25	9	5	29	45	36
MO	116	115											1	
MA	38	3	32	1			1					1		
PM	91	11		77						1			2	
CM	11				10				1	n				
iETTH	11					11								
fETTH	38						36						2	
PTTH	68						8	53					7	
CTTH	24							1	23					
CH	10			2						8				
NDPH	5										5			
MOH	28											28		
Neuralgia	26												26	
Others	65			15				6	1				7	36

*MO* migraine without aura, *MA* migraine with aura, *PM* probable migraine, *CM* chronic migraine, *iETTH* infrequent episodic tension-type headache, *fETTH* frequent episodic tension-type headache, *PTTH* probable tension-type headache, *CTTH* chronic tension-type headache, *CH* cluster headache, *TACs* trigeminal autonomic cephalalgias, *NDPH* new daily persistent headache, *MOH* medication overuse headache

The sensitivity, specificity, PPV, and NPV of the CDSS 2.0 for headache disorders from Comparison A are presented in Table 3. The results demonstrate that the system is accurate and reliable for diagnosing MO (sensitivity 89.15%, specificity 99.75%, κ = 0.920), MA (sensitivity 1, specificity 98.80%, κ = 0.908), CM (sensitivity 1, specificity 99.81%, κ = 0.951), PTTH (sensitivity 88.33%, specificity 96.82%, κ = 0.805), CTTH (sensitivity 92%, specificity 99.80%, κ = 0.936), CH (sensitivity 88.89%, specificity 99.62%, κ = 0.839), and MOH (sensitivity 96.55%, specificity 1, κ = 0.981). However, sensitivity was relatively low for PM (81.05%) and fETTH (80%), despite a very high specificity (96.79% for PM and 99.59% for fETTH). The value of κ indicated excellent agreement for PM (0.791) and fETTH (0.856). With regard to iETTH and NDPH, the system displayed perfect sensitivity and specificity (1 for both headache types).

**Table 3:** Statistical indices of CDSS 2.0 diagnostic performance in Comparison A

	Migraine				TTH				CH	NDPH	MOH	Neuralgia
	MO	MA	PM	CM	iETTH	fETTH	PTTH	CTTH				
Sensitivity	0.8915	1.0000	0.8105	1.0000	1.0000	0.8000	0.8833	0.9200	0.8889	1.0000	0.9655	0.5778
(95%CI)	0.8215–0.9372	0.8666–1.0000	0.7144–0.8809	0.6554–1.0000	0.6786–1.0000	0.6495–0.8991	0.7682–0.9479	0.7250–0.9860	0.5067–0.9942	0.4629–1.0000	0.8037–0.9982	0.4224–0.7201
Specificity	0.9975	0.9880	0.9679	0.9981	1.0000	0.9959	0.9682	0.9980	0.9962	1.0000	1.0000	1.0000
(95%CI)	0.9840–0.9999	0.9727–0.9951	0.9454–0.9816	0.9876–0.9999	0.9909–1.0000	0.9835–0.9993	0.9468–0.9814	0.9873–0.9999	0.9847–0.9993	0.9910–1.0000	0.9905–1.0000	0.9902–1.0000
False negative rate	0.0337	0	0.0409	0	0	0.0183	0.0151	0.0039	0.0019	0	0.0020	0.0376
False positive rate	0.0086	0.1579	0.1538	0.0909	0	0.0526	0.2206	0.0417	0.2000	0	0	0
Youden Index	0.8890	0.9880	0.7784	0.9981	1.0000	0.7959	0.8515	0.9180	0.8851	1.0000	0.9655	0.5778
PPV	0.9914	0.8421	0.8462	0.9091	1.0000	0.9474	0.7794	0.9583	0.8000	1.0000	1.0000	1.0000
(95%CI)	0.9459–0.9996	0.6807–0.9341	0.7519–0.9103	0.5712–0.9952	0.6786–1.0000	0.8093–0.9908	0.6594–0.8674	0.7688–0.9978	0.4422–0.9646	0.4629–1.0000	0.8498–1.0000	0.8398–1.0000
NPV	0.9663	1.0000	0.9591	1.0000	1.0000	0.9817	0.9849	0.9961	0.9981	1.0000	0.9980	0.9624
(95%CI)	0.9417–0.9807	0.9904–1.0000	0.9349–0.9749	0.9909–1.0000	0.9909–1.0000	0.9644–0.9911	0.9677–0.9934	0.9842–0.9993	0.9877–0.9999	0.9910–1.0000	0.9872–0.9999	0.9408–0.9766
kappa	0.920	0.908	0.791	0.951	1.000	0.856	0.805	0.936	0.839	1.000	0.981	0.715
(95%CI)	0.879–0.957	0.826–0.970	0.711–0.857	0.821–1.000	1.000–1.000	0.767–0.933	0.722–0.876	0.850–1.000	0.566–1.000	1.000–1.000	0.934–1.000	0.584–0.833

*MO* migraine without aura, *MA* migraine with aura, *PM* probable migraine, *CM* chronic migraine, *iETTH* infrequent episodic tension-type headache, *fETTH* frequent episodic tension-type headache, *PTTH* probable tension-type headache, *CTTH* chronic tension-type headache, *CH* cluster headache, *NDPH* new daily persistent headache, *MOH* medication overuse headache, *CI* confidence intervals, *PPV* positive predictive values, *NPV* negative predictive values

Comparison B tested the agreement between the CDSS 2.0 diagnosis and Expert Diagnosis 2, which was conducted using both auto-generated information and outpatient medical records. The distribution of headache types from Comparison B is presented in Table 4, and the statistical data are given in Table 5. The system correctly identified 111/146 (76.03%) of the patients with MO, 25/26 (96.15%) of the patients with MA, 9/10 (90%) of the patients with CM, and 64/85 (75.29%) of the patients with PM. It also correctly recognized 209/267 (78.28%) of the patients with migraine, including 157/180 (87.2%) with TTH, in whom iETTH was diagnosed in 8/9 (88.89%), fETTH in 32/44 (72.73%), CTTH in 22/23 (95.65%), and PTTH in 47/59 (79.66%). The correct recognition rates for CH, NDPH, and MOH were 77.78% (7/9), 80% (4/5), and 84.85% (28/33), respectively.

**Table 4:** Agreement between CDSS 2.0 diagnosis and Expert Diagnosis 2 in Comparison B

CDSS 2.0∖Expert	Total	MO	MA	PM	CM	iETTH	fETTH	PTTH	CTTH	CH	NDPH	MOH	Neuralgia	Others
Total	531	146	26	85	10	9	44	59	23	9	5	33	50	32
MO	116	111	1									3	1	
MA	38	8	25	1	1		1					1	1	
PM	91	18		64				4		2		1	2	
CM	11				9				1				1	
iETTH	11	1		2		8								
fETTH	38			3			32						3	
PTTH	68			3		1	8	47					8	1
CTTH	24						1		22		1		1	
CH	10			3						7				
NDPH	5			1							4			
MOH	28											28		
Neuralgia	26							1					24	1
Others	65	8		8			2	7	1				9	30

*MO* migraine without aura, *MA* migraine with aura, *PM* probable migraine, *CM* chronic migraine, *iETTH* infrequent episodic tension-type headache, *fETTH* frequent episodic tension-type headache, *PTTH* probable tension-type headache, *CTTH* chronic tension-type headache, *CH* cluster headache, *TACs* trigeminal autonomic cephalalgias, *NDPH* new daily persistent headache, *MOH* medication overuse headache

**Table 5:** Statistical indices of CDSS 2.0 diagnostic performance in Comparison B

	Migraine				TTH				CH	NDPH	MOH	Neuralgia
	MO	MA	PM	CM	iETTH	fETTH	PTTH	CTTH				
Sensitivity	0.7603	0.9615	0.7529	0.9000	0.8889	0.7273	0.7966	0.9565	0.7778	0.8000	0.8485	0.4800
(95%CI)	0.6813–0.8253	0.7842–0.9980	0.6454–0.8373	0.5412–0.9948	0.5067–0.9942	0.5696–0.8454	0.6680–0.8861	0.7603–0.9977	0.4019–0.9605	0.2988–0.9895	0.6733–0.9428	0.3388–0.6242
Specificity	0.9870	0.9743	0.9395	0.9962	0.9943	0.9877	0.9555	0.9961	0.9943	0.9981	1.0000	0.9958
(95%CI)	0.9682–0.9952	0.9552–0.9856	0.9121–0.9590	0.9846–0.9993	0.9818–0.9985	0.9720–0.9950	0.9317–0.9716	0.9843–0.9993	0.9818–0.9985	0.9877–0.9999	0.9905–1.0000	0.9834–0.9993
False negative rate	0.0843	0.0020	0.0477	0.0019	0.0019	0.0243	0.0259	0.0020	0.0038	0.0019	0.0099	0.0515
False positive rate	0.0431	0.3421	0.2967	0.1818	0.2727	0.1579	0.3088	0.0833	0.3000	0.2000	0.0000	0.0769
Youden Index	0.7473	0.9358	0.6923	0.8962	0.8832	0.7150	0.7521	0.9526	0.7721	0.7981	0.8485	0.4758
PPV	0.9569	0.6579	0.7033	0.8181	0.7273	0.8421	0.6912	0.9167	0.7000	0.8000	1.0000	0.9231
(95%CI)	0.8974–0.9840	0.4858–0.7986	0.5970–0.7922	0.4776–0.9679	0.3932–0.9267	0.6807–0.9341	0.5660–0.7946	0.7153–0.9854	0.3537–0.9191	0.2988–0.9895	0.8498–1.0000	0.7340–0.9866
NPV	0.9157	0.9980	0.9523	0.9981	0.9981	0.9757	0.9741	0.9980	0.9962	0.9981	0.9901	0.9485
(95%CI)	0.8836–0.9398	0.9869–0.9999	0.9268–0.9695	0.9876–0.9999	0.9876–0.9999	0.9567–0.9868	0.9539–0.9859	0.9873–0.9999	0.9846–0.9993	0.9877–0.9999	0.9756–0.9963	0.9245–0.9654
kappa	0.798	0.768	0.673	0.854	0.796	0.762	0.705	0.933	0.732	0.798	0.913	0.606
(95%CI)	0.733- 0.855	0.636–0.874	0.579–0.751	0.661–1.000	0.541–0.959	0.647–0.862	0.602–0.792	0.844–1.000	0.421–0.932	0.331–1.000	0.819–0.984	0.459–0.738

*MO* migraine without aura, *MA* migraine with aura, *PM* probable migraine, *CM* chronic migraine, *iETTH* infrequent episodic tension-type headache, *fETTH* frequent episodic tension-type headache, *PTTH* probable tension-type headache, *CTTH* chronic tension-type headache, *CH* cluster headache, *NDPH* new daily persistent headache, *MOH* medication overuse headache, *CI* confidence intervals, *PPV* positive predictive values, *NPV* negative predictive values

These comparative results demonstrate that the CDSS 2.0 is accurate and reliable for diagnosing MO (sensitivity 76.03%, specificity 98.70%, κ = 0.798), MA (sensitivity 96.15%, specificity 97.43%, κ = 0.768), CM (sensitivity 90%, specificity 99.62%, κ = 0.854), iETTH (sensitivity 88.89%, specificity 99.43%, κ = 0.796), fETTH (sensitivity 72.73%, specificity 98.77%, κ = 0.762), and CTTH (sensitivity 95.65%, specificity 99.61%, κ = 0.933) in an actual outpatient setting. However, diagnostic capability was relatively low for PM (sensitivity 75.29%, specificity 93.95%, κ = 0.673), PTTH (sensitivity 79.66%, specificity 95.55%, κ = 0.705), and CH (sensitivity 77.78%, specificity 99.43%, κ = 0.732). With regard to NDPH and MOH, the κ value indicated excellent agreement for these two headache types (κ = 0.798 for NDPH, κ = 0.913 for MOH). Although the sensitivity was fair (80% for NDPH, 84.85% for MOH), it had very high specificity (99.81% for NDPH and 1 for MOH).

For diagnosing neuralgia, its sensitivity was very low in both Comparison A (57.78%) and Comparison B (48%), despite a very high specificity (1 for Comparison A and 99.58% for Comparison B). The κ value indicated fair agreement for neuralgia (κ = 0.715 in Comparison A, κ = 0.606 in Comparison B).

### Results of the satisfaction survey

**Fig. 6:**
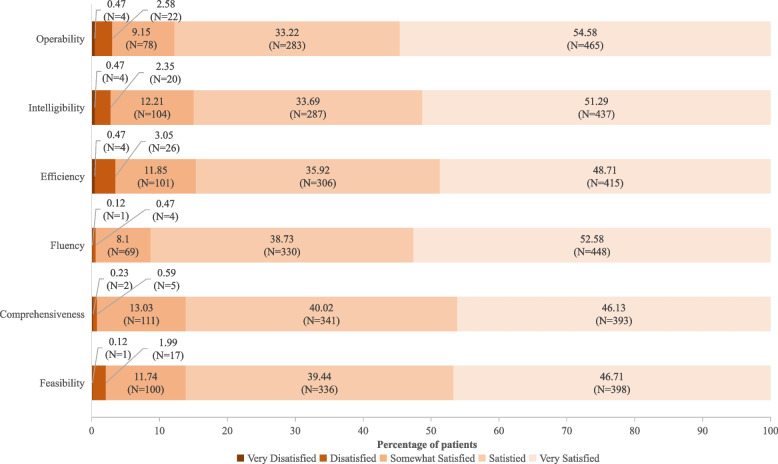
Distribution of perception and satisfaction scores in terms of understandability, usefulness, applicability, and general satisfaction (*n* = 852). The questionnaire assessed overall system satisfaction in six separate areas including operability, intelligibility, efficiency, fluency, comprehensiveness, and feasibility, respectively, with options set as very satisfied, satisfied, somewhat satisfied, dissatisfied, and very dissatisfied. Most of the patients were satisfied with the system operability (*n* = 748, 87.79%), intelligibility (*n* = 724, 84.98%), efficiency (*n* = 721, 84.62%), fluency (*n* = 778, 91.31%), comprehensiveness (*N* = 734, 86.15%), and feasibility (*n* = 734, 86.15%). Percentages may not total 100 because of rounding

At the end of the questionnaire, a satisfaction survey with seven choice questions and one open question was presented to the 852 patients. The majority of the patients (852/890, 95.73%) completed the satisfaction questionnaire, which assessed satisfaction with the use of the system during the collection of headache information. The system was very well accepted, with high levels of satisfaction reported (Fig. 6). Among the 852 patients, 86.15% (*n* = 734) considered the system to be useful overall. Most patients were very satisfied with its applicability (*n* = 748, 87.79%), understandability (*n* = 724, 84.98%), completion duration (*n* = 721, 84.62%), and running speed (*n* = 778, 91.31%). The majority of the participants considered the questions posed by the system to cover the entire picture of headache symptoms (734/852, 86.15%). A large number of patients (*n* = 738, 86.62%) declared that they would recommend this system to other headache patients. The open-ended question for additional suggestions collected 18 responses by 16 patients (1.88%). Respondents most often suggested adding more options so that the patients could add information about methods used to relieve headache, dizziness-related problems, the relationship between headache and menstruation, and sleep-related issues. By contrast, some suggested reducing the number of questions.

## Discussion

Our data indicate that the newly updated CDSS 2.0 system for diagnosing headache disorders had a high degree of accuracy in recognizing MO, MA, CM, PM, iETTH, fETTH, CTTH, PTTH, and CH. It also showed perfect recognition of NDPH and MOH. The diagnostic accuracy was very satisfactory when the specialist diagnosis was based only on the auto-generated information such that the diagnostic sensitivity and specificity for primary headache exceeded 80% and 95%, respectively. Meanwhile, when comparing the diagnosis produced by CDSS 2.0 with Expert Diagnosis 2 (original medical records combined with auto-generated information), the diagnostic sensitivity and specificity for primary headache exceeded 70% and 90%, respectively, which is still a satisfactory result.

There was a discrepancy between Comparison A and Comparison B. There are two possible reasons for this. First, in a clinical environment, the physician’s role is to interpret the linguistic data provided by the patient and to analyze the content. A diagnosis is made by applying the sets of criteria for headache disorders from the ICHD-3 to this linguistic information. Multiple elements must be assessed to make a diagnosis, and the accuracy of headache-related information is very important, such as whether the patient can accurately describe the nature, aura, and autonomic symptoms of the headache. A multitude of symptoms occur before, during, and after headache episodes, which can induce complex and heterogeneous symptoms that make it difficult for patients to describe their condition [10]. Second, even after a thorough history has been obtained, establishing a headache diagnosis according to diagnostic criteria is often an intricate process. There may be overlap between different primary headaches with simultaneous multiple symptoms, such as cranial autonomic symptoms of migraine with CH with accompanying migraine-like features [11, 12]. Such overlapping conditions are likely to be more difficult for the CDSS to accurately diagnose, particularly for headaches with cranial autonomic symptoms. A recent study that used natural language processing reported differences in lexical choices between patients with migraine and those with CH. The authors showed that machine learning algorithms have potential for classifying patient self-reported narratives of migraine or CH, with good performance [13].

The diagnostic accuracy of the CDSS 2.0, which uses information obtained through human–computer conversations, was not entirely consistent with that of CDSS 1.0 [7], particularly in terms of the sensitivity of MO (76.00% vs. 99.38%), fETTH (72.73% vs. 98.02%), CH (77.78% vs. 90.00%), NDPH (80.00% vs. 100.00%), and MOH (84.85% vs. 100.00%). By contrast, the diagnostic accuracy was comparable for MA (96.15% vs. 99.38%), CM (90.00% vs. 95.24%), and iETTH (88.89% vs. 92.31%). Although new information acquisition methods that do not require manual entry often have improved efficiency, they frequently have accuracy problems. However, in terms of the use of human–machine conversations to obtain information, the diagnostic sensitivity of the current system for PM (75.29% vs. 62.71%), PTTH (79.66% vs. 60.87%), CTTH (95.65% vs. 90.00%) was higher than the previous version of the system. In terms of specificity, the diagnostic specificity was more than 96% for most primary headaches and MOH but was slightly lower for PM (93.95%) and PTTH (95.55%). This is likely to be related to inaccuracies in the headache data obtained directly through human–computer consultation. Accordingly, further work is needed to improve the CDSS 2.0 algorithm so that the system can more easily recognize inaccurate information.

Migraine and TTH are the most common types of primary headache, and the CDSS 2.0 performed well in diagnosing these. In Comparison A, with regard to migraine subtypes, we found high sensitivity, specificity, PPV, and NPV for MO, MA, and CM, with κ = 0.9606, 1, and 0.9274, indicating excellent agreement. However, for PM, κ = 0.6978, which indicated only fair to good agreement. For the TTH subtypes, we found excellent agreement for iETTH (κ = 0.9212), PTTH (κ = 0.9757), and CTTH (κ = 0.8733). However, κ = 0.639 revealed an unsatisfactory diagnostic accordance rate for fETTH, which is likely because a proportion of fETTH was misdiagnosed as PTTH. Although there are large differences between typical migraines and TTH, the symptoms of most TTH patients are not typical, particularly in cases of TTH and migraine without aura [14]. A machine learning study identified factors for distinguishing migraine from TTH [15]. In the present study, headache specialists differentially diagnosed PM or PTTH based on their clinical experience, although there is overlap between migraine and TTH in individual patients. For example, migraines last from 4 to 72 h according to the ICHD3 criteria, but specialists are likely to diagnose patients with severe headaches that last 3 h each with migraines, particularly for patients with nausea and vomiting. These borderline clinical presentations and ill-defined boundaries may lead to errors with the CDSS. However, the algorithm has improved from the previous version (CDSS 1.0) [8], along with the diagnostic accuracy for PM (κ = 0.9212) and PTTH (κ = 0.9212). In future research, the artificial intelligence method of deep learning could be introduced to further improve the diagnostic accuracy of CDSS.

Although related studies have used CDSS technology to diagnose headache disorders, most have focused on primary types of headache such as migraine and TTH, and have not included other primary types of headache or secondary headaches. This study demonstrated that the CDSS 2.0 is a valuable tool for diagnosing headache disorders in headache clinics, both in terms of secondary and primary headache. Of the 653 recruited patients, 18.68% were considered highly suspicious of second headache by headache experts, and the proportion was close to the rate of final secondary headache (12.9%) reported previously from our headache clinic [16]. Specifically, we found that the CDSS 2.0 gave a warning regarding secondary risks for all these patients. However, the diagnosis of secondary headaches is difficult to do only by CDSS, and relative additional medical check could play an important role in correct diagnosis and discriminating diagnosis. On the other hand, the rate of significant neuroimaging abnormalities was not significant different between primary headache patients (0.58%) and healthy controls (0.73%), suggesting that neuroimaging was unnecessary for primary headache [17]. Therefore, in an actual headache clinic setting, the physician's confirmation of the information and judgment of the need for futher examination are particularly important in headache diagnosis. In terms of screening for “red flags,” our research shows that the CDSS 2.0 has the potential to warn clinicians of secondary headache with good performance. However, the final diagnosis of diagnosing secondary headache remains at the discretion of the physicians. In addition, medication overuse is a common issue in patients with primary headache disorders and can even lead to chronification of the condition, referred to as MOH, which is a specific type of secondary headache [18]. In this study, we found high sensitivity, specificity, PPV, and NPV for MOH (κ = 0.9606), indicating a satisfactory diagnostic accordance rate. Due to the higher disability and treatment specificity of MOH, CDSS2.0 gives a higher priority to MOH diagnosis (if a patient is diagnosed with both chronic migraine and MOH, CDSS2.0 would give the diagnosis of MOH). In the future, it is expected to be able to diagnose MOH and primary headache simultaneously in the upgrading of CDSS.

With regard to NDPH, which is a unique type of primary headache, the sensitivity, specificity, PPV, and NPV were all 100% when the physician entered the information into our previous version of the CDSS. In the present study, when the specialists’ diagnoses were based solely on system information, the sensitivity, specificity, PPV, and NPV were all 100%. However, one case of NDPH was misdiagnosed as CTTH when the CDSS 2.0 diagnosis was compared to the Expert diagnosis 2. This misdiagnosis may have arisen because the patient with NDPH did not understand the questions about the remembered onset of NDPH in the questionnaire. In terms of neuralgia diagnoses, the κ values indicated a less satisfactory diagnostic accordance rate. Most neuralgia diagnoses require the exclusion of secondary causes, and in this study, our system provided clues regarding secondary causes in neuralgia patients. Therefore, the CDSS 2.0 should mainly be used for the diagnosis of primary headache, such that its role in the diagnosis of neuralgia is mainly warning of secondary headache.

In the previous standalone version of the CDSS [7], clinical information was entered into the system manually by doctors. This was severely time-consuming in practical clinical applications. In this study, the efficiency of headache information acquisition was greatly improved via the patient–computer conversation format, with satisfactory diagnostic accuracy. Previous studies have attempted to increase headache diagnostic accuracy by improving the algorithm of the CDSS. For example, one study developed an end-to-end decision support system to improve the efficiency of diagnosis and follow-up for the treatment of primary headaches [19]. Considering the incompleteness of the language rules used when human experts express knowledge, a study used the Learning-From-Examples algorithm to improve correct recognition rate [20]. Another study developed a hybrid intelligent system for diagnosing primary headache disorders, applying various mathematical, statistical, and artificial intelligence techniques [21]. Although various tools have been developed to improve the diagnostic algorithm, no computer-assisted systems have obtained headache information directly from the patient. The CDSS 2.0 addresses this problem by achieving access to clinical information through human–computer conversations in an outpatient setting. Furthermore, the system was generally well accepted by patients with headache. In summary, compared to previous computerized diagnostic tools, the CDSS 2.0 has several advantages. First, to the best of our knowledge, it is the first self-administered support system for diagnosis of headache. Second, the system is web-based, supports multi-center simultaneous application, and can be used to create a data repository for further analysis. Third, it can be used to diagnose a wide range of headache types, including migraine, TTH, CH, NDPH, MOH, and neuralgia.

In fact, the objective difficulty in the management of primary headaches is the enormity of the number of patients requiring a diagnosis. However, there was important shortcomings in migraine management by general practitioners with a consequent delay in referring selected patients to dedicated headache centers [22]. Moreover, the delay in diagnosis encourages chronicity and medication overuse as a consequence of the misuse of analgesics, which further increased the burden of headache disease. Even in a high-income country with free and easily accessible headache services, headache disorders continue to be a problem. A Danish nationwide cross-sectional survey suggested almost half of individuals (43.7%) had never consulted a medical doctor for their headache; even of those with weekly headache, more than a quarter (28.3%) had never done so in their lifetimes [23]. The validity of this system lies in the possibility of offering a diagnostic tool that if offered to the general practitioner. It is believable that CDSS2.0 can help overcome the unmet needs of headache disorders by improving the diagnostic accuracy and thereby reduce the burden of headache-related conditions in China.

Nevertheless, there are several study limitations that should be addressed. First, the headache specialists made Expert Diagnosis 2 based on both the auto-generated report and out-patient medical records, rather than through direct consultation and examination. In this multi-center study, it is challenging to achieve a direct diagnosis by unified headache experts through direct history-taking for each patient. The two headache specialists consulted the physicians who directly assessed the patients when in doubt about the information in the outpatient medical records, which included detailed present history, physical examination, imaging findings, and preliminary diagnosis. Thus, the Expert Diagnosis 2 was considered close to a gold standard in this study. Then, the CDSS 2.0 focuses on headache diagnosis only, and lacks treatment recommendations. Providing patients with accurate medical advice is critical when managing headache disorders. A new web-based pharmaceutical decision support system, developed in France, effectively guides pharmacy personnel recommendations for self-medication with analgesics and identifies patients who require referrals to specialist care [6]. In addition, a previous study suggested that a Digital Migraine Tracker might aid medical communication and optimize management [24]. However, the lack of treatment and follow-up processes is a limitation of this study that should be addressed in future work. We plan to include a digitalized follow-up headache diary in the future system to track patient headaches, which could guide physician recommendations. In addition, a doctor–client component that allows physicians to revise the information entered by the patient in real time is likely to further improve the diagnostic accuracy of the CDSS 2.0.

## Conclusion

We tested and verified an updated version of our decision support system for diagnosing headache disorders, CDSS 2.0. Applying human–computer conversation, patient headache data could be efficiently collected by the system. The CDSS 2.0 demonstrated a high degree of accuracy in recognizing migraines, TTH, CH, NDPH, and MOH but not neuralgia. Given the high diagnostic accuracy for most of the primary headache types and a satisfactory ability to alert for secondary headaches, this system is likely to improve diagnostic accuracy for headache disorders and thereby reduce the burden of headache-related conditions in China. In addition, the CDSS 2.0 was well accepted by patients, and adding a doctor–client component and follow-up processes will be future directions for expansion and improvement. We expect that these changes will enhance the diagnostic ability of the system.

## Supplementary Information

**Additional file 1.** Satisfaction Survey Questions.

## Data Availability

The datasets used and/or analysed during the current study are available from the corresponding author on reasonable request.

## Abbreviations

CDSS: Clinical decision support system
MO: Migraine without aura
MA: Migraine with aura
CM: Chronic migraine
PM: Probable migraine
iETTH: Infrequent episodic tension-type headache
fETTH: Frequent episodic tension-type headache
CTTH: Chronic tension-type headache
PTTH: Probable tension-type headache
CH: Cluster headache
NDPH: New daily persistent headache
MOH: Medication overuse headache
YLD: Years lived with disability
TTH: Tension-type headache
ICHD-3: The third edition of the International Classification of Headache Disorders
PHQ-9: The Patient Health Questionnaire-9
GAD-7: Generalized Anxiety Disorder Scale
VAS: Visual analog scale
PPV: Positive predictive values
NPV: Negative predictive values
CI: Confidence intervals
SEM: Standard error of the mean
