# Supplementary material for: Verification of a clinical decision support system for the diagnosis of headache disorders based on patient–computer interactions: a multi-center study
Source: J Headache Pain. 2023 May 23;24(1):57. doi: 10.1186/s10194-023-01586-1 (PMC10204238; doi:10.1186/s10194-023-01586-1)
Supplement: Supplementary file 1 — Additional file 1. Satisfaction Survey Questions. [file 10194_2023_1586_MOESM1_ESM.docx]

**ADDITIONAL FILE 1**

**Satisfaction Survey Questions**

1) The system operates smoothly without much instruction. (5-point Likert scale)

2) The descriptions used in the system are well understood and do not require much explanation. (5-point Likert scale)

3) The system took me a moderate amount of time to fill out the questionnaires, and it did not occupy me too much time. (5-point Likert scale)

4) The system runs smoothly. (5-point Likert scale)

5) The questionnaires collected covered the whole picture of my headache. (5-point Likert scale)

6) Do you think the system is useful as a whole? (5-point Likert scale)

7) Do you proceed to recommend this system to other headache patients? (yes/no)

8) Do you have any remarks or suggestions? (open-ended)
